# A Newly-Identified Polymorphism in Rhesus Macaque Complement Factor H Modulates Binding Affinity for Meningococcal FHbp

**DOI:** 10.1371/journal.pone.0135996

**Published:** 2015-08-18

**Authors:** Monica Konar, Peter T. Beernink, Dan M. Granoff

**Affiliations:** Center for Immunobiology and Vaccine Development, UCSF Benioff Children’s Hospital Oakland, Oakland, California, United States of America; Emory University School of Medicine, UNITED STATES

## Abstract

**Background:**

Two meningococcal serogroup B vaccines contain Factor H binding protein (FHbp). Binding of Factor H (FH) to FHbp was thought to be specific for human or chimpanzee FH. However, in a previous study an amino acid polymorphism in rhesus macaque FH domain 6, tyrosine at position 352 (Y352) was associated with high binding to FHbp, whereas histidine at position 352 (H352) was associated with low binding.

**Methods and Results:**

Here we report that a second FH polymorphism at position 360 also affects macaque FH binding. Of 43 macaques, 11 had high FH binding and 32 had low binding. As in our previous study, all 11 animals with high binding had Y352, and 24 with low binding had H352. However the remaining eight with low FH binding had Y352, which was predicted to yield high binding. All eight had S360 instead of P360. Thus, three allelic variants at positions 352 and 360 affect macaque FH binding to FHbp: HP (low), YS (low), and YP (high). We measured binding affinity of each FH sequence type to FHbp by surface plasmon resonance. Two animals with high binding types (YS/YP and HP/YP) had dissociation constants (*K*_*D*_) of 10.4 and 18.2 nM, respectively, which were similar to human FH (19.8 nM). Two macaques with low binding (HP/HP and HP/YS) had *K*_*D*_ values approximately five-fold higher (100.3 and 99.5 nM, respectively). A third macaque with low binding (YS/YS) had a *K*_*D*_ value too high to be measured.

**Conclusions:**

Macaques have at least three allelic variants encoding FH with different affinities for FHbp (five genotypic combinations of these variants). Since in previous studies binding of FH to FHbp vaccines decreased protective antibody responses, our data will aid in selection of macaques with FH binding that is similar to humans for further investigation of FHbp vaccine immunogenicity.

## Introduction

Factor H binding protein (FHbp) is an important antigen in two recently licensed meningococcal serogroup B vaccines (Bexsero, Novartis Vaccines and Diagnostics; and Trumenba, Pfizer Inc.). FHbp binds human complement Factor H (FH), which down-regulates complement activation and allows the organism to evade complement [1]. Early studies reported that binding of FH to FHbp was specific for human and chimpanzee FH [2]. Further, in immunized human FH transgenic mice, binding of FH to FHbp vaccines decreased protective anti-FHbp antibody responses [3–6], and mutant FHbp vaccines with decreased FH binding elicited serum antibodies with higher protective titers [3–5, 7] (reviewed in [8, 9]).

Human FH contains 20 domains and domains 6 and 7 are responsible for binding of human FH to FHbp [10]. Recently FH from a subset of rhesus macaques from the California National Primate Research Center was reported to bind to FHbp with a similar affinity as that of human FH [11]. In macaque FH, a single amino acid polymorphism in domain 6, tyrosine at residue 352 (Y352) was associated with high binding to FHbp, whereas histidine (H352) was associated with low binding to FHbp. In the present study, we describe a second macaque FH polymorphism at residue 360 that can interact functionally with residue 352 and affect FH binding. We further characterized the kinetics and affinities of different FH sequence types for binding of macaque FH to FHbp.

## Materials and Methods

### Ethics statement

The experiments in non-human primates were performed in strict adherence to the "Guide for the Care and Use of Laboratory Animals" [12] and the Weatherall report, "The Use of Non-human Primates in Research" (http://www.acmedsci.ac.uk/download.php?f=file&i=13211). Animals were kept in outdoor social housing with their dams and extended families, were under the care of experienced veterinarians, and only minimally-invasive procedures were performed. The macaques received weekly produce, daily foraging mix (sunflowers and corn), and opportunistic browsing in the form of trees and branches. Each cage was fitted with A-frames, hanging barrels and small mirrors affixed to the sides of the cage. Some of the cages also had play structures, grass and high perches. The dimensions of the outdoor cages were 100 x 200 ft. The study was approved by the Institutional Animal Care and Use Committee at the University of California, Davis (Protocol No. 18217). Human serum samples used as controls for measuring FH binding and affinity were obtained with written informed consent under a protocol approved by the Institutional Review Board at Children’s Hospital & Research Center Oakland (Protocol No. 2002–010).

### Role of sponsor

The results reported in this manuscript were from studies supported by grants from the National Institute of Allergy and Infectious Diseases, National Institutes of Health, and the California National Primate Research Center (to D.M.G. and/or P.T.B.). The funders had no role in the study design, data collection and analysis, decision to publish, or preparation of the manuscript.

### Blood and serum samples

Blood and serum samples were obtained from 43 rhesus macaques, ages 2 to 3 months, cared for at the California National Primate Research Center. One additional was in our previous study of macaque polymorphisms affecting binding of FHbp [11]. This animal was included in the present study because of a distinctive phenotype (see Results and Discussion section). Data on FH polymorphisms for the remaining 42 macaques have not been previously reported. Fifteen of the animals, however, were described in a recent vaccine immunogenicity study [13].

### Binding of FH to FHbp by ELISA

We assayed binding of serum FH to FHbp by ELISA, which was performed as previously described [5]. Briefly, the wells of a microtiter plate were coated with recombinant FHbp ID 1 (2 μg/ml in PBS; 100 μl per well), which was purified as described previously [14] with the addition of a second ion exchange chromatography step (HiTrap SP HP; GE Life Sciences). The plate was incubated at 4°C overnight. After washing and blocking, two-fold serial dilutions of macaque sera were added to the wells starting at a dilution of 1:100. After incubation for 2 h at room temperature, bound FH was detected with a sheep anti-human FH antibody (1:7,000; Abcam). The bound sheep IgG was detected with donkey anti-sheep IgG conjugated to alkaline phosphatase (AP) (Sigma-Aldrich; 1:5,000) for 1 h at room temperature. The assays were performed in three independent experiments, each in duplicate and every plate had the same human serum sample as a positive control.

### DNA sequencing

Genomic DNA was isolated from blood of each of the animals using the DNeasy Blood and Tissue Kit (Qiagen). The genes encoding FH domains 6 and 7 were amplified using Phusion high-fidelity DNA polymerase (Thermo Scientific) and primers described previously [11]. PCR products were purified using the PCR Purification Kit (Qiagen) and quantified by measuring the absorbance at 260 nm (NanoDrop 1000; Thermo Scientific). DNA sequencing was performed by a commercial service (Sequetech) using the forward and/or reverse PCR primers.

### Cloning of FH domain 6

To determine FH amino acid sequences encoded by each allele for the two animals that were heterozygous at both positions (i.e., Y and H at residue 352 and P and S at residue 360), we amplified and cloned domain 6 exons and performed DNA sequencing. The exons were amplified using Phusion high-fidelity DNA polymerase (Thermo Scientific) and the PCR products were incubated with *Taq* DNA polymerase and dNTPs at 70°C for 10 min to introduce deoxyadenosine overhangs at the 3’ ends, and were cloned into pGEM-T-Easy (Promega). The plasmid clones were transformed into *Escherichia coli* DH5α (Invitrogen). Plasmid DNA was prepared (Plasmid Miniprep Kit, Qiagen) and ten plasmid clones from each of the two macaques were subjected to DNA sequencing with the T7 primer.

### Surface plasmon resonance (SPR)

Kinetics and affinities of the different macaque FH amino acid sequence types for binding to FHbp were determined by SPR using a Biacore X100 Plus instrument (GE Life Sciences). We used a capture assay to purify serum FH on the biosensor chip and measure binding of solution-phase FHbp. We exploited a single-cycle kinetics approach because insufficient volumes of individual macaque sera were available to allow purification of FH from each serum sample. Further, the converse approach of using immobilized FHbp, produced responses from interactions with other serum proteins and confounded the quantitative analysis of FH interactions.

In brief, 5,000 to 9,000 response units of goat anti-human FH (Complement Technologies) that had been affinity purified on a human FH column were immobilized to a CM5 biosensor chip (GE Life Sciences) using the Amine Coupling Kit (GE Life Sciences). FH (~600 RU) was captured from a 1:50 serum dilution and increasing concentrations of FHbp ranging from 3.16 to 316 nM were tested. Data were analyzed with Biacore X100 Evaluation software (GE Life Sciences) using a 1:1 binding model. Each serum sample was analyzed in two independent experiments, each performed in duplicate or triplicate. To ascertain the specificity of the FH capture experiments for macaque FH, as opposed to FH related proteins, we performed Western blotting with the capturing antibody and did not detect binding of macaque or human serum proteins other than FH. As an additional control, we compared the binding kinetic parameters of human serum FH and human FH purified from the same subject, which were similar to each other.

## Results and Discussion

**Fig 1:**
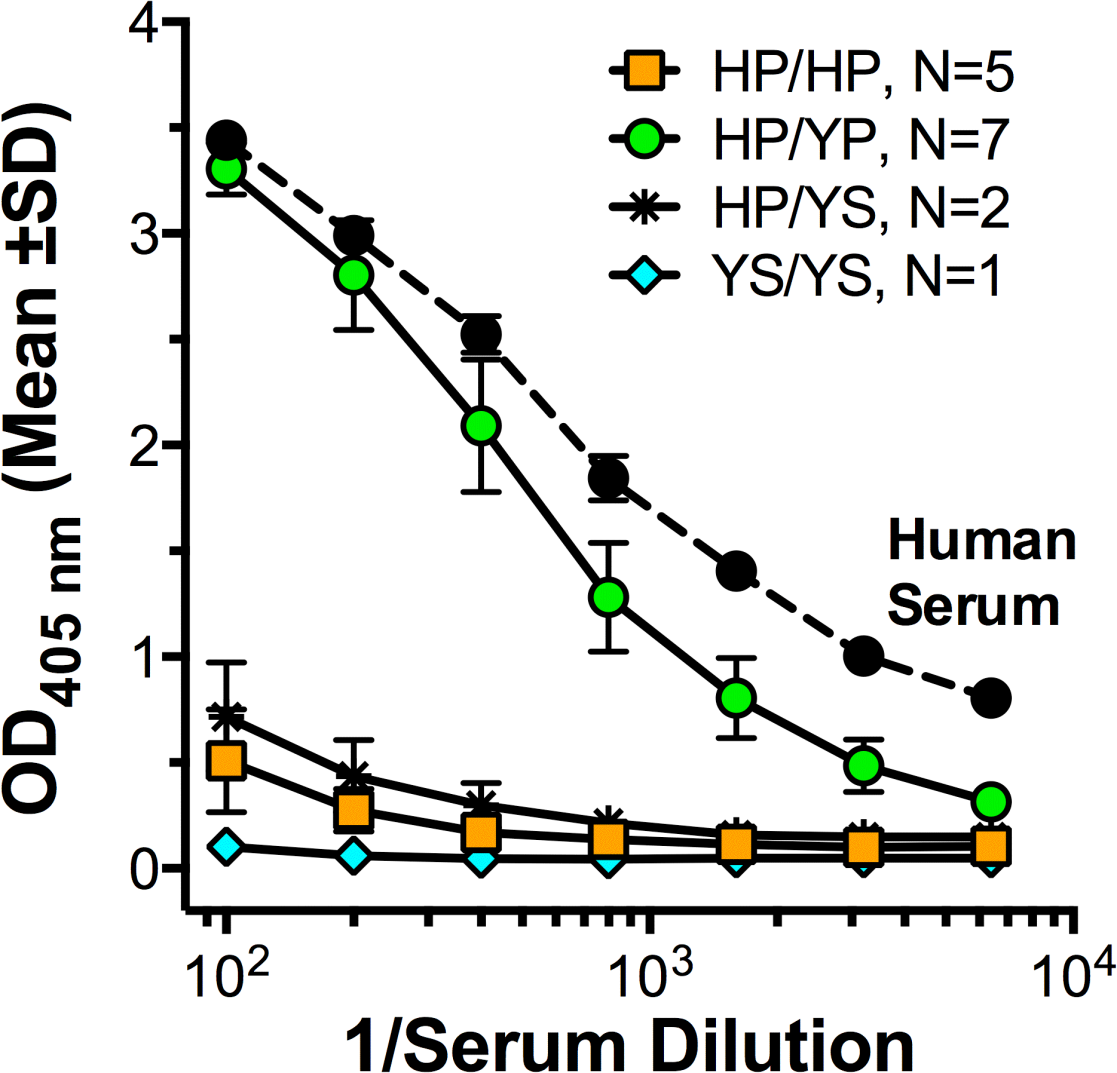
Binding of serum FH to FHbp by ELISA. Representative data (mean ±SD) from 7 macaques with high serum FH binding to FHbp and eight with low binding. For comparison, FH binding in a control human serum is shown (filled black circles). Green circles, animals heterozygous in FH domain 6: H352 with P360 (HP) and Y352 with P360 (YP). Orange squares, animals homozygous for H352 with P360 (HP/HP); blue diamonds, an animal homozygous for Y352 and S360 (YS/YS); asterisks, animals heterozygous for HP/YS (see **Table 1**).

As described in the Introduction section, in previous studies binding of FH to FHbp was reported to be specific for human or chimpanzee FH [2], and for FH from a subset (~30%) of rhesus macaques. Of 43 macaques in the present study, 11 macaques had high FH binding to FHbp as measured by ELISA, and 32 had low FH binding. Representative binding data for 15 of the animals are shown in **Fig 1**, and the results for all 43 animals are summarized in Table 1.

**Table 1:** Polymorphisms in FH domain 6 for rhesus macaques with high or low binding of FH to FHbp.

Number of Rhesus Macaques	Serum FH Binding to FHbp	Macaque Factor H Domain 6		
		Amino Acid Position 352	Amino Acid Position 360	Amino Acid Sequence Type (Positions 352 and 360)
24	Low	H	P	HP/HP
1	Low	Y	S	YS/YS
7	Low	H and Y	P and S	HP/YS^a^
1^b^	High	Y	P and S	YS/YP
10	High	H and Y	P	HP/YP

^**a**^For animals with H and Y at 352, and P and S at 360, each residue pair listed (e.g. HP) is encoded by an allele as inferred from DNA sequence analysis of cloned exons from two animals with this sequence type.

^b^Animal reported to have Y352 in a previous study [11]. We had not detected the P and S polymorphism at 360 in the previous study but on closer inspection of the sequence traces found this polymorphism in one of the 14 animals tested.

In our previous study [11], the DNA sequences encoding FH domain 7 were identical for all 14 animals. In FH domain 6, all seven macaques with high FH binding were heterozygous for Tyrosine (Y) and histidine (H) at residue 352, and all seven macaques with low FH binding were homozygous for H at residue 352. In the present study the DNA sequences encoding FH domain 7 also were identical for all 16 macaques tested. For FH domain 6, the 11 animals with high FH binding to FHbp were either homozygous Y (N = 1) at residue 352 or were heterozygous Y and H (N = 10, Table 1). The results from this group were consistent with our previously reported results [11]. In the present study, 24 of the animals with low FH binding were homozygous for H352, which was also consistent with our previous data. However, the remaining 8 animals with low FH binding had Y352, which, based on our previous data, was predicted to be high FH binding. All 8 of these animals had S360 instead of P360 (Table 1). Thus, three allelic variants at positions 352 and 360 affect binding of macaque FH to FHbp: HP (low), YS (low), and YP (high) (**Table 1**).

**Fig 2:**
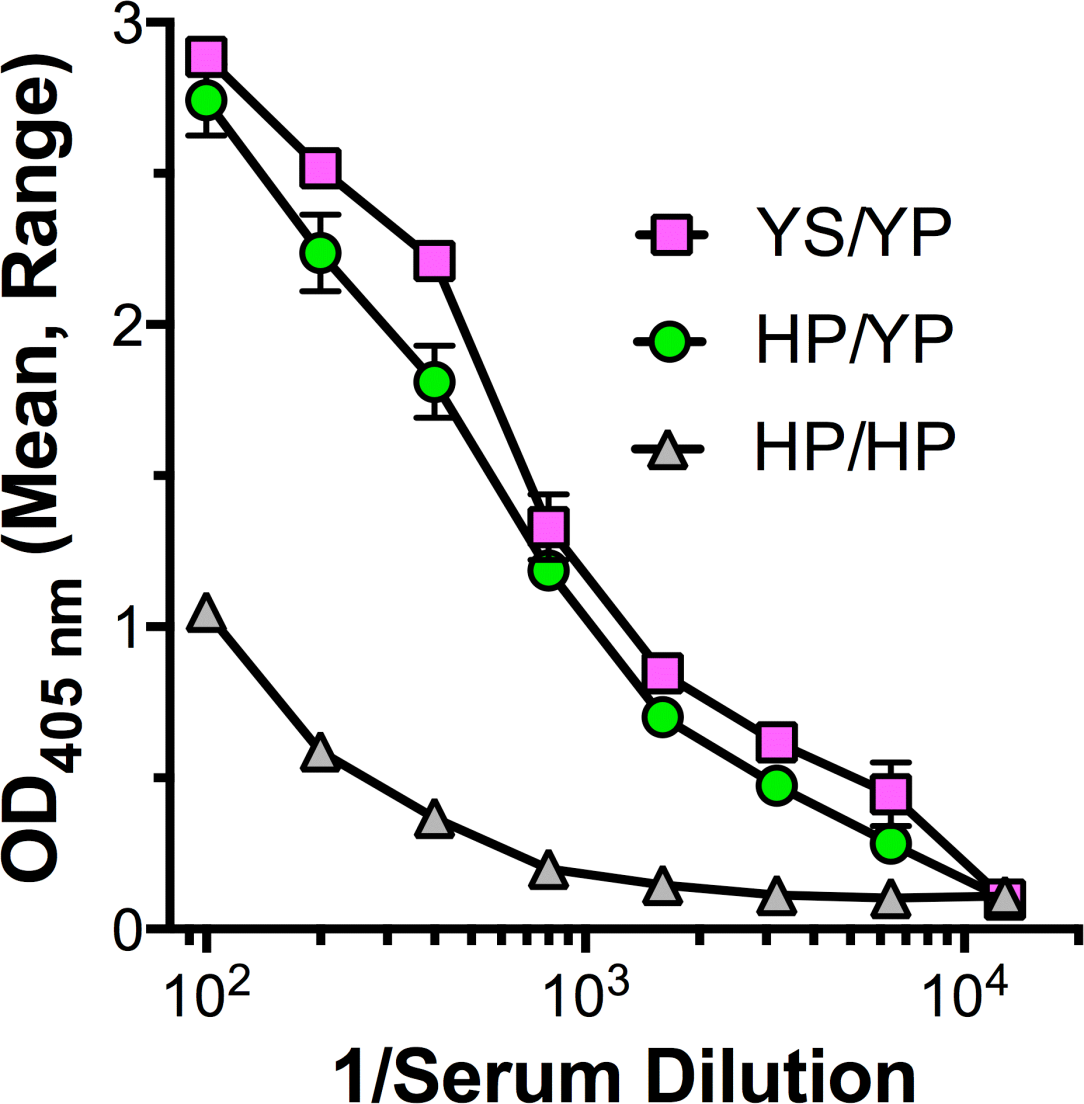
Binding of macaque serum FH to FHbp by ELISA. An animal with high FH binding to FHbp was heterozygous in domain 6 for Y352 with S360 (YS) and Y352 with P360 (YP) (magenta squares). For comparison, examples are shown of a high FH binding control serum from an animal heterozygous with H352 and P360 (HP) and Y352 and P360 (YP) (green circles), and a control serum with low FH binding from an animal with homozygous H352 and P360 (HP/HP) (grey triangles). The means and ranges of two to four replicates are shown.

To confirm the combination of polymorphisms encoded by each allele, we amplified the exons encoding FH domain 6, cloned the products and sequenced 10 clones derived from each of the two animals. In all of the individual clones from the two heterozygous animals, H352 always was present with P360, and Y352 always was present with S360. An additional animal heterozygous with Y352,P360 and Y352,S360 had a high binding phenotype (**Table 1**). Collectively, the data indicate that the combination of Y352 with P360 is dominant with respect to high binding (**Fig 2**).

We also measured the affinity for binding of macaque serum FH to FHbp by surface plasmon resonance (SPR) for several representative animals with high or low FH binding to FHbp (**Table 2**). Two of the high binding macaque sera (macaques 44342 and 41941) had dissociation constants (*K*_*D*_) of 18.2 and 10.4, respectively, which were similar to those measured for human serum FH (19.8 nM). Two of the low binding macaque sera (macaques 44322 and 44333) had *K*_*D*_ values that were five- to 10-fold higher (99.5 and 100.3 nM) than the macaques with high binding (note, the higher the *K*_*D*_, the lower the affinity). The affinity of a FH from a third macaque (44370) with low binding was too low to be measured. Collectively the ELISA and SPR data indicated that the FH containing Y352,S360 allele had lower binding to FHbp than FH containing the H352/P360 allele, which was also associated with low binding affinity to FHbp.

**Table 2:** Kinetic parameters for binding of macaque FH to FHbp.

			SPR Parameter, Mean ±SE^a^				
Serum	FH Sequence Type (Residues 352 and 360)^b^	ELISABinding	*K*_*D*_ (M) x 10^9^	*k*_*a*_ (M^-1^ s^-1^) x 10^−6^	*k*_*d*_ (M^-1^) x 10^3^	R_max_ (RU)	χ^2^ (RU^2^)
Macaque 44322	HP/HP	Low	100.3 ±24.8	0.18 ±0.01	19.0± 0.3	22.9 ±2.2	0.60 ±0.26
Macaque 44333	HP/YS	Low	99.5 ±18.2	0.19 ±0.02	18.8± 0.5	14.3 ±0.5	0.35 ±0.08
Macaque 44370	YS/YS	Low	—-^c^	—-^c^	—-^c^	3.5	—-^c^
Macaque 44342	HP/YP	High	18.2 ±0.9	0.18 ±0.01	3.18 ±0.16	27.3 ±2.0	1.56 ±0.32
Macaque 41941	YS/YP	High	10.4 ±0.3	0.18 ±0.02	1.89 ±0.21	42.3 ±4.4	2.24 ±0.63
Human	ND	High	19.8 ±0.5	0.19 ±0.01	3.82 ±0.02	36.4 ±0.2	0.87 ±0.12
Human^d^	ND	High	18.2 ±0.9	0.22 ±0.02	4.11 ±0.69	44.0 ±3.8	1.84 ±0.27

^a^ The mean and standard error (SE) are shown for a total of five replicates performed in two independent experiments. *K*_*D*_, equilibrium dissociation constant; *k*_*a*_, association rate constant; *k*_*d*_, dissociation rate constant; R_max_ is the maximal binding response and Chi^2^ (χ^2^) is the quality of fit of a 1:1 binding model to the data. When the χ^2^ value was ~5% of the R_max_ value or less, the fit was considered reliable.

^b^ Amino acids at positions 352 and 360 for each of the two alleles inferred from DNA sequencing of FH domain 6

^c^—, macaque with YS/YS had R_max_ too low to determine kinetic parameters

^d^ Purified FH from same human subject

**Fig 3:**
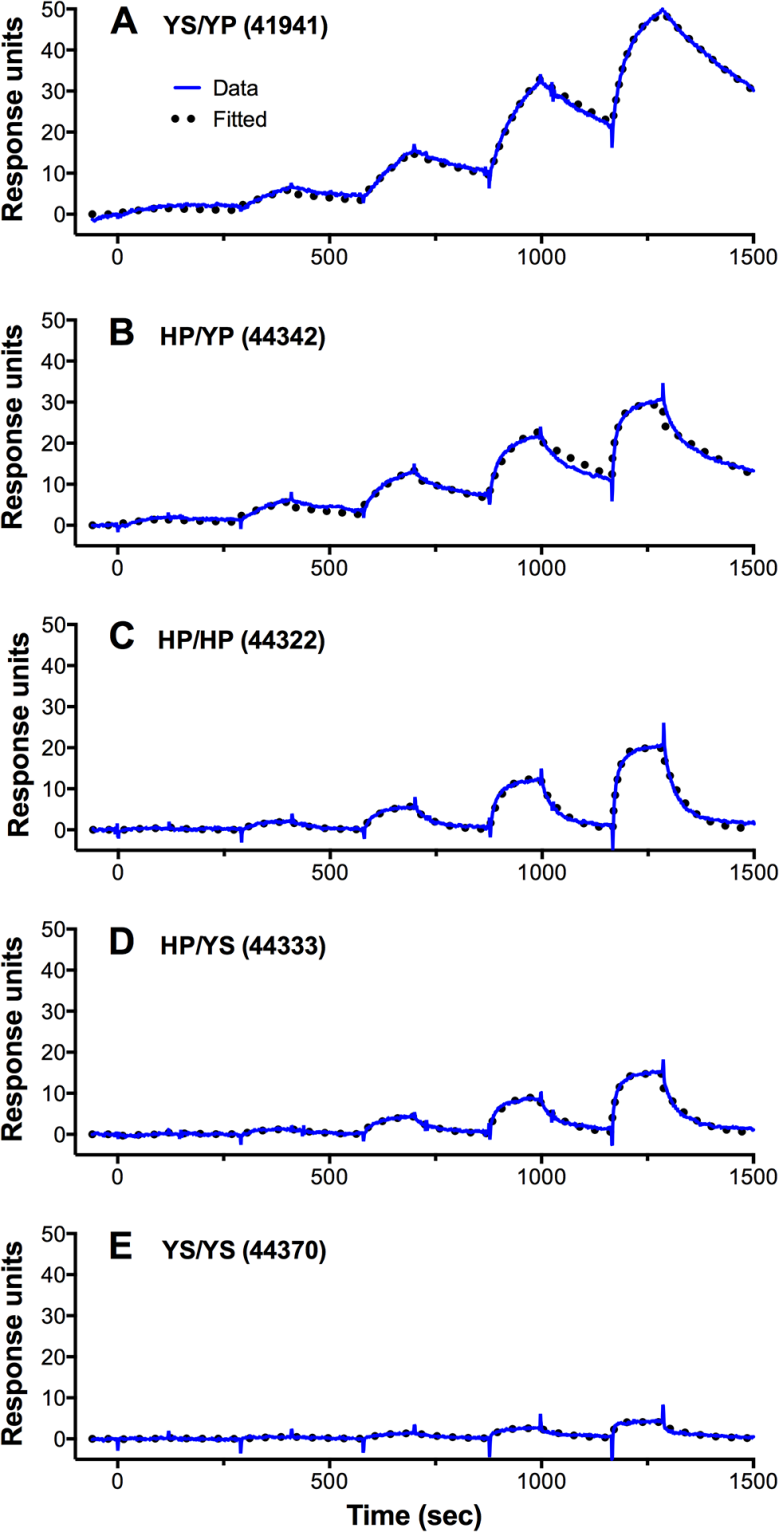
Surface plasmon resonance data for binding of meningococcal FHbp to captured macaque FH. Macaque FH was captured using an immobilized anti-human FH antibody and binding kinetic parameters were determined in single-cycle kinetics experiments as described in Methods. Data were analyzed with Biacore X100 Evaluation software (GE Life Sciences) and plotted in Prism 6.0e (GraphPad, Inc.). Representative data for one of five replicates performed with serum from one animal with each FH sequence type are shown.

Interestingly the respective association rate constants (*k*_*a*_) were similar for animals with high or low FH binding, whereas the dissociation rate constants (*k*_*d*_) varied by as much as 10-fold (**Table 2**). Representative SPR binding data and least squares fits to the data for all five macaques are shown in **Fig 3**.

**Fig 4:**
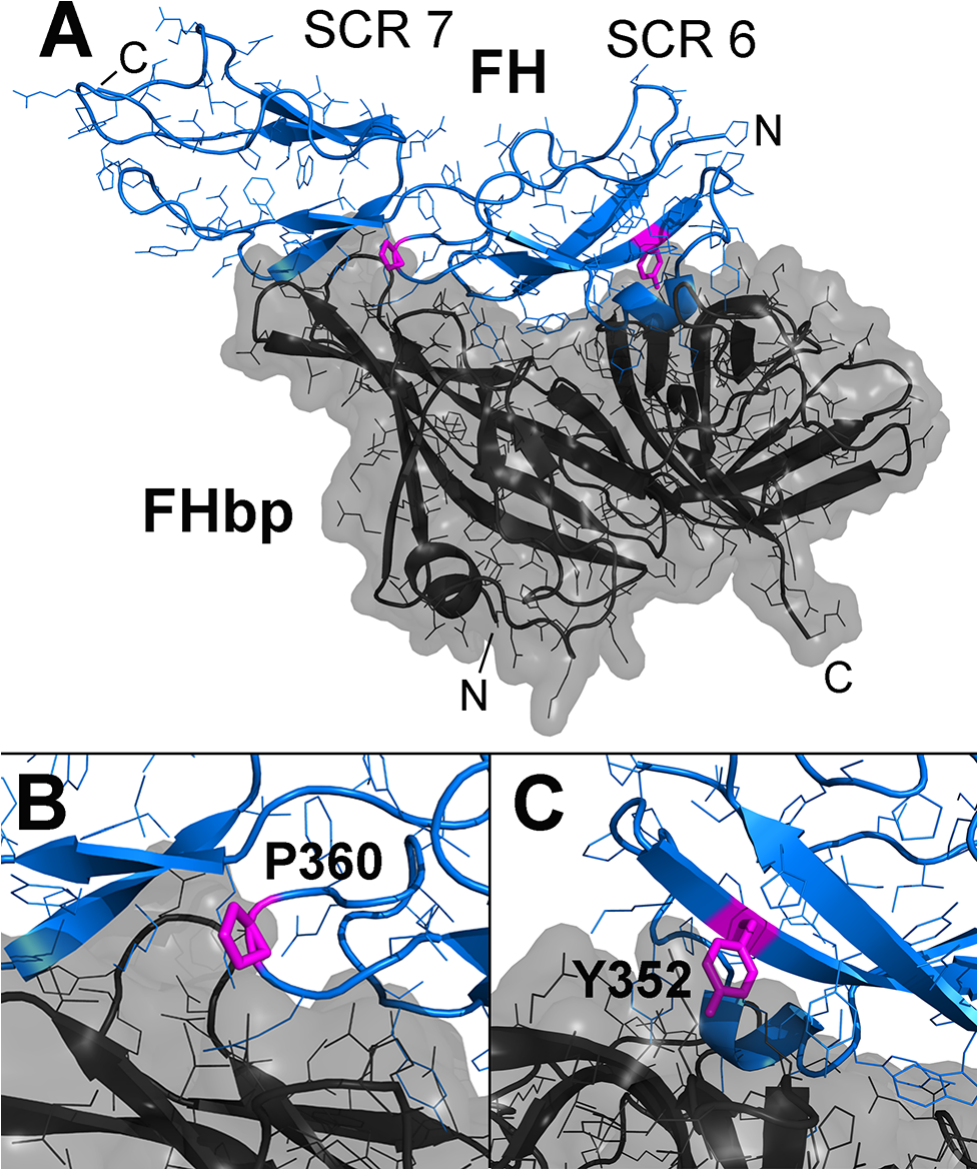
Homology model of macaque FH domains 6 and 7 bound to FHbp. **A.** Structure of modeled complex between macaque FH (blue) and FHbp (dark grey). The FH residues that are the sites of polymorphisms are shown (magenta). FH short consensus repeat (SCR) domains 6 and 7 are labeled. **B.** Closer view of the region of P360. **C.** Closer view of Y352. The homology model was constructed with Swiss Model [16] using the structural coordinates of the FHbp-FH fragment complex [10].

Based on a structural homology model of macaque FH domains 6 and 7 bound to FHbp derived from the complex with human FH [15], FH residues 352 and 360 both are in proximity to FHbp (**Fig 4A**). Proline 360 is at the end of a loop of FH that lies in a cleft of FHbp and is a histidine in human FH (**Fig 4B**). Tyrosine 352 in FH projects into a cleft of FHbp and is present in human FH (**Fig 4C)**. Thus polymorphisms at either of these two positions likely independently affect binding of macaque FH to FHbp through direct effects. We have observed three different macaque FH alleles encoding two low binding variants, HP and YS, and one high binding variant, YP. Although we have observed two different residues at each of the two positions (H or Y at 352 and P or S at 360), we have not observed the combination of H352 with S360.

We also found that for different macaque FH sequence variants from homozygous animals, HP binds with higher affinity than YS (**Fig 1; Table 2**). Further, comparing HP/YP to HP/HP animals indicates that YP binds with higher affinity than HP. Since the YS/YS sequence type has no detectable binding either by ELISA or SPR, our hypothesis is that in measuring the affinity of YS/YP for FHbp there is little or no contribution of the YS variant. As a result, the YS/YP variant yields a higher affinity (lower *K*_*D*_, 10.4 nM) than the animals with HP/YP, in which both variants have measurable binding. Thus the measured *K*_*D*_, 18.2 nM, in the animal with HP/YP likely reflects binding of a mixture of high and low affinity variants.

Binding of FH decreases the protective antibody responses of human FH transgenic mice immunized with FHbp vaccines [9]. Since binding of FHbp was thought to be specific for human and chimpanzee FH [2], and studies using chimpanzees are not practical, investigating the effect of FH binding on FHbp vaccine immunogenicity could not be extended to other non-human primates, which would likely be more relevant to human immunogenicity than transgenic mouse model. The results of the present study of polymorphisms affecting binding of macaque FH to FHbp therefore will be useful for selecting animals to investigate the effect of binding of FH on FHbp immunogenicity and for evaluating protective antibody responses elicited by mutant FHbp antigens with low binding of FH, which are expected to translate into more effective, and possibly safer, FHbp vaccines for humans [8, 9].
